# Exploring the Relationship between WeChat Usage and E-purchase Intention During the COVID-19 Pandemic Among University Students in China

**DOI:** 10.1177/21582440221139447

**Published:** 2022-11-25

**Authors:** Haitham Medhat Abdelaziz Elsayed Aboulilah, Syed Far Abid Hossain, Bui Nhat Vuong, Tawfiq Jebril

**Affiliations:** 1 School of Management, Xi’an Jiaotong University, China; 2 BRAC Business School, BRAC University, Dhaka, Bangladesh; 3 Ton Duc Thang University, Vietnam

**Keywords:** WeChat, e-purchase, COVID-19, university, students, China

## Abstract

Young generations, especially students, are increasingly turning their attention to e-purchasing apps. However, little has been investigated regarding students’ tendencies during market turbulence or a pandemic situation such as COVID-19. To address this knowledge gap, this study develops a model from the perspective of e-purchase intention for university students during the COVID-19 pandemic based on one of the most famous social network sites (SNSs), WeChat, in China. The model is tested using survey data from 608 students studying in China. The results indicate that WeChat, as a popular and commonly used social media, affects users in their e-purchase intention during the COVID-19 pandemic in China through information shared by various users. Further, the effect of trust moderates the relationship between market turbulence and e-purchase intention among university students in China. Despite some limitations, such as survey data collected from students only in a single country, the study contributes to theory and practice by shedding light on SNS-based e-purchase intention among students in China during market turbulence. Theoretical contributions and managerial implications gleaned from this study and its empirical results are discussed.

## Introduction

The novel coronavirus infection and the subsequent global crisis was testified in Wuhan, China, in December 2019 for the first time (Xu, Gutierrez et al., 2020), later spreading exceedingly rapidly across the globe. The outbreak subsequently affected economic systems and businesses severely. Without an open innovation system (Chesbrough, 2019), finding a rapid solution to ensure regular sales has become extremely difficult recently. According to a report published in CNN, 90% of students in the world are affected due to lockdown (CNN, 2020). China has followed a strict lockdown policy since 23rd January 2020 (Lu-Hai, 2020); since 15th April, some people have started to return to the workplace. However, the lockdown policy seems stricter for university students. From 3rd May 2020, some universities began to allow their students to return with certain conditions. Many students were locked down in the dormitory or rented apartment in which they were studying. During this time, it was literally impossible to buy anything from traditional shops or markets. The only way to buy necessary items was through e-purchase.

In China, WeChat is the most popular method of communication and payment, with 1,0825 billion monthly active subscribers (Tencent, 2019). University students are connected to each other, communicate with each other, and engage with each other for various activities via WeChat (Chen, 2017). WeChat is unique among social networking platforms because of its safe payment and user satisfaction (Wen et al., 2016). There are numerous e-sellers who promote and sell various goods and services independently via WeChat as a platform (Hossain, Nurunnabi et al., 2020; Hossain, Xi et al., 2020). Some students consider this work as a trajectory of part-time income and try to sell things within their own communication channels (Hossain, 2019). During COVID-19, students have been more reliant on social networking to obtain information about the outbreak. At the same time, it has been observed that they are using WeChat as a tool to buy their necessary daily goods. Despite the initial lack of availability of certain types of products in the market, such as hand wash, facial masks, hand sanitizer, etc., WeChat was able to offer, through e-shopping, a way to find these items at affordable price during the outbreak. Recent research has observed WeChat selling behavior from different perspectives such as social capital (Chen, Ma et al., 2021), gratification analysis (Gan & Li, 2018), supply chain management (Yan et al., 2018), educational management (Baloch et al., 2021; Khoshaim et al., 2020; Far Abid Hossain et al., 2020), etc.; however, research on WeChat shopping tendency during COVID-19 is lacking due to the recent occurrence of the coronavirus outbreak.

This research aims to address the above-mentioned research gap and make numerous contributions to the existing e-shopping literature. First of all, the study recognizes the distinctive connection between e-shopping tendency and WeChat usage among university students in China during the COVID-19 outbreak. Second, it clarifies the reasons behind using WeChat as a tool or platform in this pandemic situation. Lastly, it encompasses the existing literature by discovering users’ intention to continue using WeChat in the future. In the subsequent sections, a theoretical model describing the hypothesized relationships is developed with research methodology and further analysis and results, limitations, and conclusion.

## Relevant Literature, Theoretical Background, and Hypotheses Development

In the pre-COVID-19 stage, the pattern of online purchase behavior varied. Scholarly articles investigated consumer information sharing and e-WOM as a significant mediator for the fashion and clothing industry (Rahman & Mannan, 2018). In the e-commerce platform, the consumers behave inversely based on segmentation analysis (Huseynov & Özkan Yıldırım, 2019). This strange type of behavior is the outcome of trust and belief (Hossain, Nurunnabi et al., 2020). However, during the COVID-19 pandemic, buyers had fewer options to think and verify. The necessity for obtaining the desired product on time was more crucial. As a result, the previous studies, such as variety-seeking tendency (Hossain et al., 2019) in the pre-COVID-19 stage, contradict this study. This contradiction is not for the perceived satisfaction but for the emergency. Previous studies investigated market turbulence (Terawatanavong et al., 2011); gratification analysis (Gan & Li, 2018); social network sites (Wang & Chen, 2012); however, the changing pattern in buying behavior of the students with a social media platform (WeChat) received less attention among the scholars. Thus, the phenomenon is still under the shadow. In this study, we attempted to explore it in the next few sections.

### Self-Presentation During Market Turbulence

The technological acceptance model (TAM), initially developed by Davis (1989), has been widely accepted and used with the technology adoption process. The theory holds the understanding that, through perceived helpfulness and perceived ease of use, it is possible to effectively determine the behavioral intentions that may lead to the actual usage of any technology, such as WeChat. The behavioral intention has been determined as a key indicator of user acceptance of any technology, including apps. According to previous theoretical contributions, TAM is a powerful tool of analysis that has received much attention from scholars due to its robustness. To determine individual behavioral intention toward technology usage, TAM has also been utilized by the leading managers (Grandon & Pearson, 2004). Self-presentation is a psychological phenomenon that arises among specific individuals who are either seeking to help others or want to attract social attention via social networking. During market turbulence, firms try to use innovativeness to meet or exceed their regular sales targets (Tsai & Yang, 2013). At the same time, some independent users or virtual sellers may attract buyers’ attention. If the buyers are satisfied and subsequently share this information by posting on their social network, this may attract attention from other potential buyers, especially during market turbulence such as COVID-19. Due to self-presentation, a potential customer may be aware of the effect of market turbulence (Terawatanavong et al., 2011) and act accordingly. An example is feeling the need to purchase health and safety products during COVID-19 due to self-presentation. Thus, we hypothesize:

*H1. Self-presentation (via WeChat) positively influences market turbulence (such as COVID-19) in terms of e-purchase intention.*

### Information Seeking During Market Turbulenceand E-purchase Intention

During COVID-19, the information-seeking scenario is different. The current theory states that anxiety can reduce information-seeking tendency (Soane et al., 2015); however, during COVID-19, due to difficulties in production because of the absence of labor or transportation issues, buyers are facing product shortages in the market (Kampf et al., 2020). As a result, the information-seeking tendency has increased among potential buyers to obtain their desired products. WeChat is an easily accessible platform that enables users to seek information online in China. WeChat users frequently share information among mutual friends (Zhang et al., 2017), which may lead to further online purchasing based on information sharing such as pictorial presentations, the sharing of video clips, short advertisements, the sharing of product specifications, and the sharing of personal experience regarding a certain product or service, all of which can be quickly and easily shared via WeChat among users’ circles of friends (Wang et al., 2018). In this way, during COVID-19, customers who are usually not considered as online information seekers in relation to purchasing are also engaged in e-purchasing. Although some people only consider buying services online, such as tour packages (Zhu et al., 2019), people in general are observed to be seeking information online during COVID-19 in China. More specifically, students living alone, away from their parents, have had no alternative to e-purchasing due to lockdown issues (CNN, 2020). Thus, we hypothesize:

*H2*. Information seeking (via WeChat) positively influences market turbulence (such as COVID-19).
*H3.* Information seeking (via WeChat) positively influence e-purchase intention.

### Social Interaction During Market Turbulenceand E-purchase Intention

Due to advanced technological development, social interaction has gained much attention among researchers regarding knowledge sharing and general practices among particular networks or groups of known people (Abdallah, 2020). Due to market turbulence, such as COVID-19, social-interaction tendency increases, due in this case to lockdown issues. Researchers have discovered that social interaction aids learning through effective social networks (Al-Hasan, 2021). WeChat is considered as an effective tool in China in terms of social network systems or platforms where people not only communicate information but can also buy and sell goods and services. Current literature shows increased social-interaction tendency due to effective social network sites (SNSs) (Wang & Chen, 2012). As marketing in terms of network economy is being increasingly studied and understood by researchers (Achrol & Kotler, 1999), the role of social interaction is increasingly being considered both strong and meaningful in times of market turbulence when a potential customer is struggling to make a purchase decision. The increased e-purchase intention is observed due to increased media-entrepreneurship tendency (Hossain, 2019). This indicates that social interaction through an SNS platform like WeChat can increase e-purchase intention.

Thus, we hypothesize:

*H4*. Social interaction (via WeChat) positively influences market turbulence (such as COVID-19).
*H5*. Social interaction (via WeChat) positively influences e-purchase intention.

### The Power of SNS Comments During Market Turbulence and E-purchase Intention

The traditional concept of word of mouth (WOM) has been replaced with electronic WOM (e-WOM) due to developments in the social networking arena (Loureiro & Kaufmann, 2020). Usually, buyers read online comments to learn more about a product or service before making a final purchase decision. Online engagement depends on positive or negative comments given by other users or customers (Loureiro & Kaufmann, 2020). During a pandemic situation, an economic downturn is, historically, an extremely common scenario (Mandal, 2020). As a result, customers or buyers usually spend less due to the uncertainty of their income. On the other hand, the opposite scenario has also been observed among some customers who buy excessive amounts of consumer products (Misra & Adewumi, 2020) (sometimes referred to as panic buying) during an emergency or outbreak situation such as a natural disaster or a pandemic. Comments may be influential in this context by motivating or demotivating buyers to make a purchase intention. During COVID-19, some people have purchased large amounts of products, especially facial masks, hand sanitizer, hand soap, etc. However, in social media comments, this has been discouraged, and people have been requested not to create a market shortage, or artificial product shortage. Therefore, we hypothesize that:

*H6.* Comments (via WeChat) positively influence market turbulence (such as COVID-19).

### Buyers’ E-purchase Intention During Market Turbulence

Technologies facilitate buyer-seller coordination and help to disseminate information (Weber, 2001), especially during market turbulence such as COVID-19. Traditional purchasing has often been replaced by e-purchasing for numerous reasons. First, product supply is not adequate in the usual outlets (markets or shopping centers), and businesses are worried both about the product supply and unexpected buyer behavior (Foster et al., 2019). In this circumstance, either firms or intermediaries try to reach customers via alternative routes. Second, market turbulence is considered a business opportunity for some independent virtual e-sellers. Regarding buyer search behavior, researchers have discovered recently alternative purchasing strategies among buyers (Foster et al., 2019). Third, the strict lock-down policy has reduced the opportunity for physical shopping tendency, especially in an infection-related pandemic situation, and motivated people to switch to e-purchasing. In this circumstance, e-sellers have been observed to adopt innovative strategies in the face of market turbulence that may influence online purchase behavior (Roy et al., 2019). Therefore, we hypothesize that:

*H7.* Market turbulence (such as COVID-19) positively influences e-purchase intention.

### Moderating Role of Trust

The authors explore how the direct relationship among the variables in the study may vary, depending on a changing environment such as COVID-19. Specifically, we theorize that trust invigorates the relationship between market turbulence (such as COVID-19) and e-purchase intention. First, we hypothesize a positive interaction effect between market turbulence (such as COVID-19) and e-purchase intention. According to recent research, salespeople may influence the purchase decision through various tactics that may be moderated by trust (Hartmann et al., 2020). In particular, trust and knowledge sharing mediate the relationship between buyers and sellers (Rungsithong & Meyer, 2020), especially in an emergency situation such as the COVID-19 outbreak. The possible reasons for this could be increased anxiety and decision-making problems, due to trust in, and mobile payment on Talwar et al. (2020), apps such as WeChat. Due to this moderating effect, e-purchase intention can either increase or decrease during market turbulence. As buyers become less dependent on e-purchase intention, the moderating effect increase. Therefore, we hypothesize that:

*H8*. Trust moderates the relationship between market turbulence (such as COVID-19) and e-purchase intention.

## Research Methodology

### Data Collection

According to scholarly articles, general respondents are less biased when it comes to decide demographic statistics of the respondents (Soror et al., 2015). However, based on the theme and context of this study, university students are used as respondents. The target population for this study included university students studying in Shaanxi province, China, who are usually familiar with WeChat or relevant social media platforms to buy online. Initial data were collected from different Universities in Shaanxi province, China resulting in 619 responses, 11 of which were discarded due to incomplete answers. Thus, after careful review of the collected data, 608 respondents remained for analysis. The study used a set of questionnaires with a 5-point likert scale and distributed to the expected respondents on WeChat and other social media platforms with convenience sampling technique during the period April-May 2020. The target respondents were active current university students who are studying in Shaanxi province, china. The specific area was selected due to a large number of universities. The questionnaires were distributed through e-mails, WeChat, and different social media networks. Due to COVID-19, no face-to-face data collection was conducted due to safety measures. Data of the study were collected with the help of some administrative staff and student representatives. The respondents were requested to participate in the survey on a voluntary basis without offering any compensation. However, a small amount of random lucky money (WeChat red packet) was offered, and the respondents were free to accept or reject the remuneration. To meet the ethical standards, the respondents were requested to answer the questions on a voluntary basis without bias. Privacy was strictly maintained during and after the data collection process to ensure ethical consideration for the respondents.

Table 1 represents the summary of the respondents’ demographic statistics. Table 1 below represents the demographic statistics of the respondents, where the majority of the respondents were male (71.9%), and they are full-time students (92.6%). They are from different educational backgrounds, and most of them are experienced in using WeChat (95.2%).

**Table 1.** Demographic Statistics.

*N* = 608	Frequency	Percentage
Gender		
Female	171	28.1
Male	437	71.9
Age group		
18–22	217	35.7
23–27	191	31.4
28–32	140	23.0
32+	60	9.9
Study major		
Business/Management	293	48.2
Science/Engineering	187	30.8
Arts/Social Science	65	10.7
Medical	32	5.3
Others	31	5.1
Full-time student		
No	45	7.4
Yes	563	92.6
Educational level		
Bachelor	265	43.6
Masters	191	31.4
PhD	118	19.4
Others	34	5.6
WeChat usage		
No	29	4.8
Yes	579	95.2

### Measurement

Each of the scales developed for building the questionnaire were adapted from preceding instruments. Furthermore, the expression was adapted for this study to reproduce its focus on WeChat. The authors tried to confirm the supreme steadiness with earlier research. The scales ranged from 1 (strongly disagree) to 5 (strongly agree). The authors designated the instruments based on all aspects of the research, with a substantial amount of time spent on the scale development.

All the measurement items for our constructs have demonstrated high significant reliability and validity according to the previous evidence. The authors used items for self-presentation developed by Tseëlon (1992) while the items for Information seeking were all adapted from Ku et al. (2013). Items for comments were adapted from de Matos and Rossi (2008). Social interaction scales were adapted from Liu et al. (2016) and Papacharissi (2002). Three scales of Market turbulence were adapted from Calantone et al. (2003). Three scales of Trust were adapted from Harris and Goode (2010) and three scales of e-purchase Intention were adapted from Putrevu and Lord (1994).

### Model Fit

**Figure 1.**
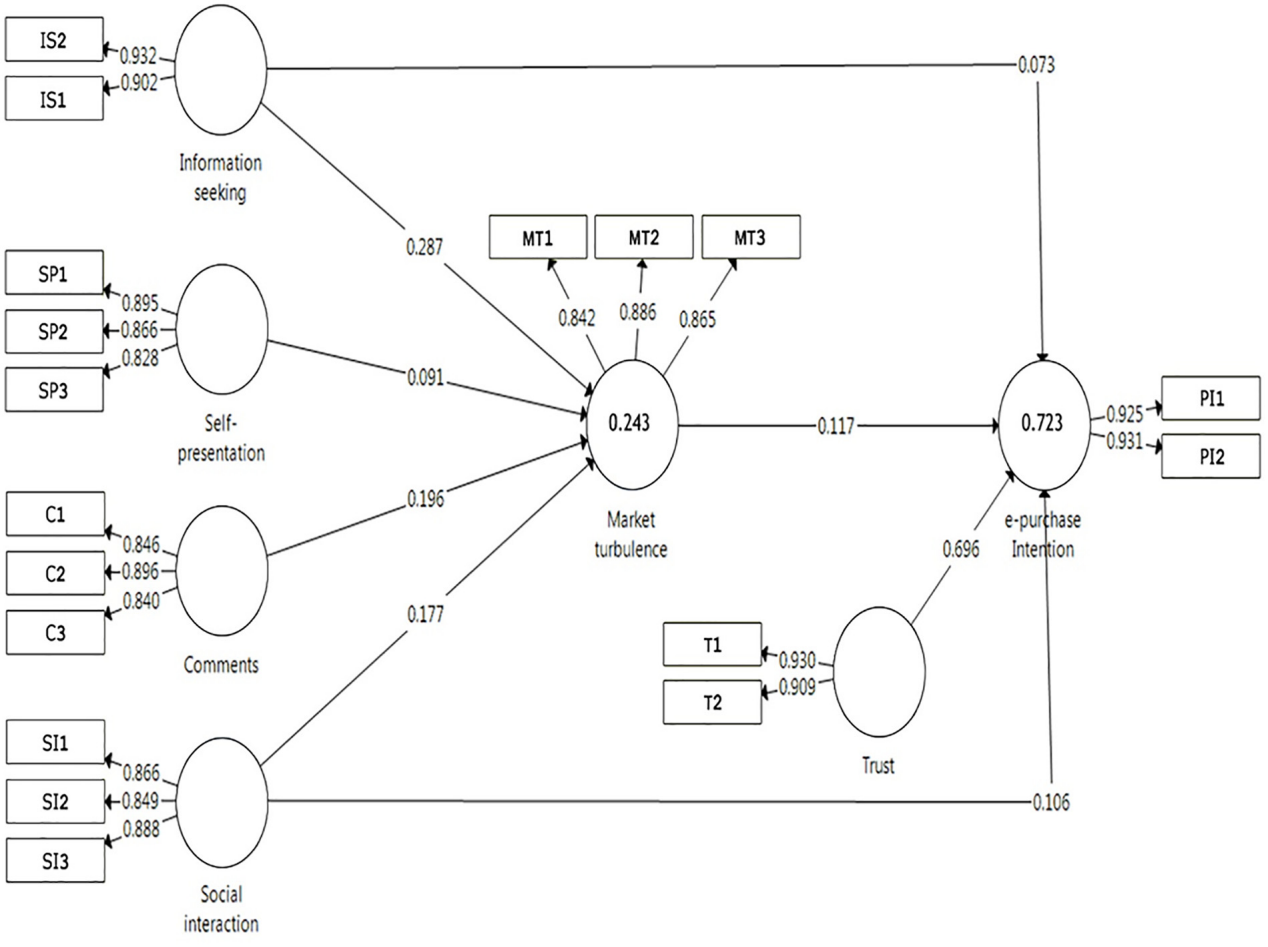
Measurement model.

The predictive power of model is evaluated by assessing the coefficient of determination (R^2^), where scores range from 0 to 1 and indicate the amount of variance explained in the dependent construct. The R^2^ values range from 0.02 (weak), to 0.13 (moderate), and 0.26 and above (substantial). “The higher the R^2^ coefficient, the better the construct is explained by the latent constructs in the structural model. The high R^2^ coefficient also reveals that the values of the variables can be well predicted by the PLS path model” (Hair et al., 2012). The R^2^ coefficient for the e-purchase intention was 0.723, it showed that 72.3% of the variation of e-purchase intention might be explained by information seeking, self-presentation, comments, social interaction, and market turbulence (Figure 1). Evaluation of the results showed substantial values, with the best results from the full sample. Thus, this research demonstrated a good model-data fit.

### Partial Least Squares Regression

The authors used the partial least squares structural equation modeling (PLS-SEM) to test the stated hypotheses. PLS-SEM was selected based on the ability to produce results from small samples (e.g., sample size less than 50) and the ability to handle non-normal data (Hair et al., 2012). Moreover, PLS-SEM can calculate complicated issues on different variables, including testing of indirect relationships, compound moderation analysis. Therefore, additional goodness of fit test like the covariance-based structural equation model was not required (Garson, 2016) in this study. For expected result with PLS, the latent variables should be in a consistent form. As the value of latent variables is a consequence of linear combinations of the indicator variables, those indicator variables should be standardized. After standardization, the path coefficients are from 0 to ±1, with the path that is the nearby to 1 being the robust (Garson, 2016). With SmartPLS 3.0, raw data is the anticipated input, as normalization is spontaneously applied, ensuing in indicator weights and latent variable scores being consistent. Therefore, in this study, SmartPLS 3.0 was utilized for data analysis.

## Research Results

Vuong and Suntrayuth (2020) suggested that the construct reliability and validity should be examined before evaluating PLS-SEM estimation for hypotheses testing. Internal consistency reliability uses Cronbach’s alpha and composite reliability as estimates of reliability. Composite reliability and Cronbach’s alpha and values need to be above the .70 critical threshold. However, composite reliability values above 0.90 indicate multiple measures of the same thing and are not desirable Hair et al. (2012). Convergent validity is supported when each item has an outer loading above 0.70 and the average variance extracted (AVE) for the variable is 0.50 or higher (Fornell & Larcker, 1981). The results of the convergent validity assessment showed the data to be highly related, which is expected and not expected to cause issues with analysis. For example, the minimum outer loading for Information seeking = 0.902, Self-presentation = 0.828, Comments = 0.846, Social interaction = 0.849, Market turbulence = 0.842, Trust = 0.909, and e-purchase intention = 0.925. Besides, the constructs’ AVE were ranging from 0.742 to 0.862 (comments and e-purchase intention, respectively) (Table 2). Therefore, all variables indicated good convergent validity.

**Table 2.** Reliability, Validity Results of Constructs.

	CA	CR	rho_A	AVE	C	IS	MT	SP	SI	T	PI
C	0.829	0.896	0.873	0.742	(0.861)	0.342	0.354	0.005	0.340	0.425	0.486
IS	0.813	0.914	0.829	0.841		(0.917)	0.394	0.020	0.217	0.483	0.478
MT	0.831	0.899	0.831	0.748			(0.865)	0.083	0.297	0.456	0.494
SP	0.836	0.898	0.895	0.746				(0.864)	−0.089	0.093	0.116
SI	0.837	0.902	0.852	0.753					(0.868)	0.460	0.476
T	0.819	0.917	0.828	0.846						(0.920)	0.833
PI	0.839	0.926	0.840	0.862							(0.928)

Square roots of AVE of latent constructs were shown in the parentheses.

CA = Cronbach’s alpha; CR = Composite Reliability; AVE = Average Variance Extracted; C = Comments; IS = Information seeking; MT = Market turbulence; SP = Self-presentation; SI = Social interaction; *T* = Trust; PI = e-purchase Intention.

Discriminant validity identifies and measures the degree that items in a specific construct are different from other constructs, to ensure the distinctiveness of the item to construct measurement. This measure is determined by establishing correlations of the constructs by comparing the square root of the AVE of a particular construct, resulting in diagonal loadings being greater than their vertical counterparts (Fornell & Larcker, 1981). The Fornell and Larcker criterion results for the full sample (Table 2) indicated no problem with discriminant validity. The square root of the AVE of each construct (in the parentheses) was verified that it was greater than the value of the construct’s highest correlation with any other construct. For example, the AVE coefficient of Market turbulence was .748, and the square root of its AVE was 0.865. This value coefficient was greater than the correlation coefficients in its column (.354 and .394) and its row (.083, .297, .456, and .494). Hence, discriminant validity for the variables was proven.

Additionally, the composite reliability and Cronbach’s alpha values were employed to evaluate reliability. The composite reliability values range between 0.896 and 0.926, which are all higher than the minimum value of 0.7 as outlined by Hair et al. (2012). Cronbach’s α values ranged from .813 to .839, above the recommended .7 value (Hair et al., 2012). Moreover, Dijkstra and Henseler (2015) suggested that “the rho_A coefficient is the important reliability measure for the partial least squares.” The rho_A value should be higher than 0.7 (Hair et al., 2012). Table 2 showed that the rho_A values range from 0.828 to 0.885. Thus, the reliability of all constructs was verified.

Henseler et al. (2016) suggested that “using heterotraitmonotrait ratio of correlations (HTMT) is necessary to confirm discriminant validity for PLS.” According to Vuong and Khanh Giao (2020), discriminant validity between the two constructs will be verified when the HTMT ratio is lower than 1.0. Table 3 indicated the Heterotrait-Monotrait Ratio values of each of the variables were below 0.70. Thus, the discriminant validity of constructs was confirmed for HTMT70.

**Table 3.** Heterotrait-Monotrait Ratio (HTMT).

	C	IS	MT	SP	SI	T	PI
C							
IS	0.424						
MT	0.412	0.478					
SP	0.048	0.065	0.102				
SI	0.403	0.260	0.349	0.112			
T	0.511	0.584	0.550	0.111	0.552		
PI	0.584	0.574	0.592	0.132	0.563	0.431	

C = Comments; IS = Information seeking; MT = Market turbulence; SP = Self-presentation; SI = Social interaction; *T* = Trust; PI = e-purchase intention.

Finally, assessing the PLS-SEM structural model begins with an assessment of collinearity issues. Collinearity issues are evaluated by variance inflation values (VIF) for the inner model values. VIF values below 5 are acceptable (Hair et al., 2012). However, other recommendations exist for evaluating VIF values and suggest that values of above 4 do not void the results of regression analysis (Vuong & Khanh Giao, 2020). Table 4 displayed the inner VIF values for the full sample. The maximum inner VIF coefficient of variables was 1.692. Overall, the collinearity issues were not a concern.

**Table 4.** Multi Collinearity Statistic.

	Market turbulence	E-purchase intention
Comments	1.237	
Information seeking	1.148	1.373
Market turbulence		1.347
Self-presentation	1.010	
Social interaction	1.157	1.285
Trust		1.692

**Figure 2.**
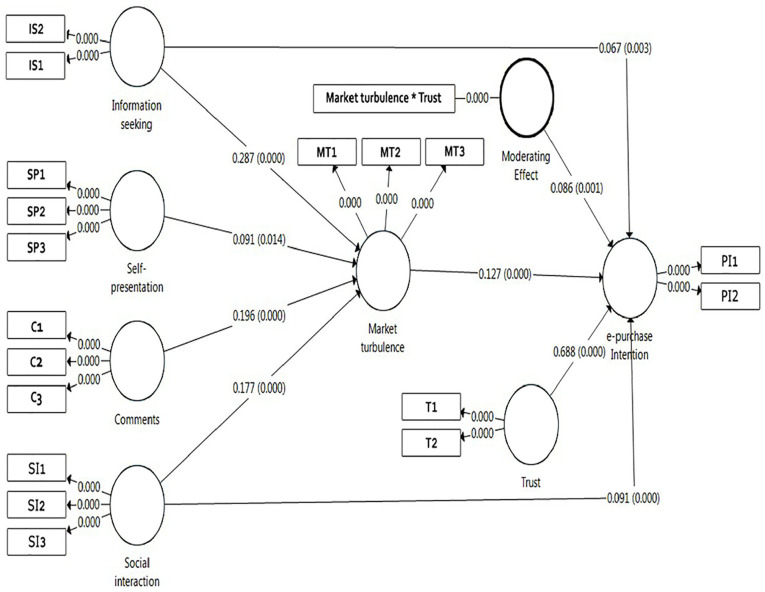
PLS-SEM model.

After running the bootstrapping procedure, t-values were evaluated to examine the statistical significance of the coefficient. A complete illustration of results derived from the structural model is demonstrated in Figure 2.

Hypothesis 1: the result exhibited that information seeking had a positive and significant relationship with market turbulence (beta coefficient = .287 and *p* = .000) (Table 5). The findings suggested that the more information seeking, the more the possibility that it will have high degrees of market turbulence. Therefore, Hypothesis 1 was supported.
Hypothesis 2: the result discovered that information seeking had a positive impact on e-purchase intention with the *p* = .003 and the standardized coefficient of .067, which recommended that information seeking is one of the preliminary components predicting consumer’s e-purchase intention. So, Hypothesis 2 was supported.
Hypothesis 3: This study found that self-presentation had a positive and significant connection with market turbulence (beta coefficient = .091 and *p* = .014) (Table 5). Thus, Hypothesis 3 was supported.
Hypothesis 4: The findings revealed that comments had a positive impact on market turbulence with the *p* = .000 and the standardized coefficient of .196, which states that comments strongly offer individuals make e-purchase intention. So, Hypothesis 4 was supported.
Hypothesis 5 and 6: The outcomes displayed that social interaction had a positive influence on market turbulence and e-purchase intention with the standardized coefficients of .177, .091, respectively, and *p* = .000. Therefore, Hypothesis 5 and 6 were supported.
Hypothesis 7: The findings confirmed that market turbulence had a positive influence on e-purchase intention with the *p* = .000 and the standardized coefficient of .127. Thus, Hypothesis 6 was supported.

**Table 5.** Hypothesis Testing.

Hypothesis	Relationship	Path coefficient	Standard deviation	T-statistics	*p*-Values	Result
H1	IS → MT	0.287	0.038	7.475	.000	Supported
H2	IS → PI	0.067	0.022	2.997	.003	Supported
H3	SP → MT	0.091	0.037	2.470	.014	Supported
H4	C → MT	0.196	0.043	4.588	.000	Supported
H5	SI → MT	0.177	0.039	4.582	.000	Supported
H6	SI → PI	0.091	0.022	4.074	.000	Supported
H7	MT → PI	0.127	0.026	4.860	.000	Supported

C = Comments; IS = Information seeking; MT = Market turbulence; SP = Self-presentation; SI = Social interaction; *T* = Trust; PI = e-purchase Intention.

### The moderating role of trust

**Figure 3.**
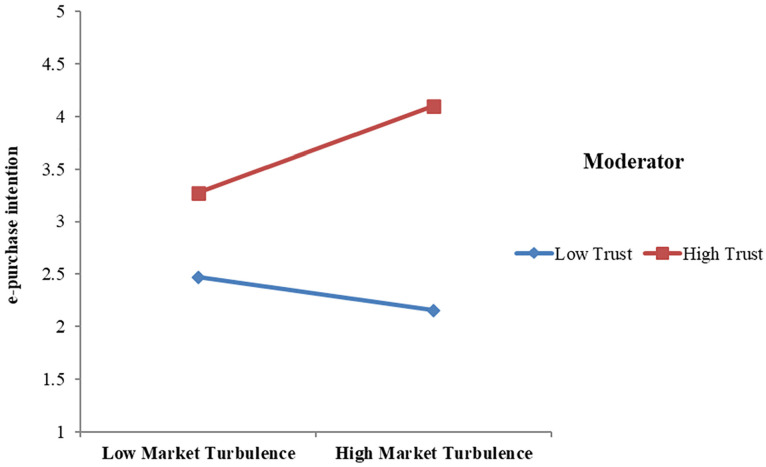
Trust strengthens the positive relationship between market turbulence and e-purchase intention.

Hypothesis 8 predicted that trust would moderate the association between market turbulence and e-purchase. The research revealed that the moderating effect of the interaction between market turbulence and trust was positive and statistically significant (beta coefficient = .086 and *p* = .001) (Table 6). This finding proposed that trust positively moderated the relationship between market turbulence and e-purchase. Besides, market turbulence positively impacted e-purchase (Hypothesis 7). Thus, the positive relationship between market turbulence and e-purchase was stronger for customers with high trust in e-commerce (Figure 3). Therefore, Hypothesis 8 was supported.

**Table 6.** The Result of the Moderating Effect.

Hypothesis	Relationship	Original sample	Standard deviation	T-statistics	*p*-Values	Moderating effect
H8	MT → PI	0.127	0.026	4.860	.000	Supported
	T → PI	0.688	0.025	27.162	.000	
	Moderating effect → PI	0.086	0.025	3.418	.001	

*Note.* Moderating effect = MT * T.

## Discussion and Conclusion

### Main Findings

This study has investigated how WeChat, as a popular and commonly used social media, affects users’ e-purchase intention during the COVID-19 pandemic in China by extending the TAM theory (Davis, 1989). The authors conceptualize technology (apps) use for e-purchase intention in the novel situation of the coronavirus outbreak. By conducting regression and moderation analysis based on a survey from China during the COVID-19 epidemic, our results validate the theory. The study explored whether there is a significant positive relationship between WeChat usage and e-purchase intention during COVID-19 among university students in China. Consistent with past research indicating that the SNS sellers, such as those who sell via WeChat (Hossain, Nurunnabi et al., 2020), can positively influence online customers during a pandemic situation. Our results show that self-presentation, customers’ online comments, information seeking, and information sharing are directly associated and affected due to market turbulence. We empirically demonstrate that trust plays a moderating role in this regard and significantly affects e-purchase intention. This finding is consistent with our theoretical arguments that, through perceived usefulness and perceived ease of use, it is possible to effectively determine the behavioral intentions that may lead to the actual usage of any technology, such as WeChat. In addition, we found that market turbulence has a direct effect on e-purchase intention. This is aligned with prior findings showing that, during market turbulence, firms attempt to use trust to meet or exceed their regular sales targets (Tsai & Yang, 2013). Our results further indicate that there is a significant positive relationship between WeChat usage and e-purchase intention during COVID-19 among university students in China due to the ability to use the same app for information gathering and purchase decisions simultaneously.

### Theoretical Implications

This study offers numerous significant theoretical contributions. First, in response to the TAM model (Davis, 1989), the use of WeChat in relation to e-purchase intention extends the theory by discovering new ways in which technology can be accepted (WeChat is a popular app that combines the benefits of an SNS and mobile banking) during an outbreak or epidemic situation in China when most financial institutions and even shopping centers have been closed. The findings confirm prior research (Foster et al., 2019) in terms of the behavioral demand effect. This study hence sheds light on the critical issue of the relationship between WeChat usage and e-purchase intention during COVID-19 among university students in China. Specifically, in this study, we found that there is a significant positive relationship between WeChat usage and e-purchase intention during COVID-19 among university students in China. The adaptation and enrichment of WeChat usage are new, from a theoretical perspective, due to the novel COVID-19 outbreak. Second, this study deepens understanding of SNS usage during an emergency as a purchasing platform, both as a means of purchasing essential items and as a means of accessing information shared by others in the same media. The study reveals that WeChat is a unique app in this regard, as it is a means of information sharing, which affects e-purchase intention, and also has a secure instant payment option. Third, this study adds to IS theory, as e-purchasing has never been so easy on one platform during an outbreak period such as COVID-19. We also explicate the moderating role of “trust” by uncovering its interaction effects with e-purchase intention. Finally, this study contributes to the current theory by studying e-purchase intention tendencies via WeChat during a pandemic situation in China.

### Managerial Implications

The findings of this research also reveal numerous managerial implications from a pragmatic viewpoint. The theoretical model of this study, therefore, represents SNSs as a tool for e-shopping, with trust as a moderating factor. The high level of information seeking and information sharing on social media such as WeChat leads to e-purchase decisions being affected instantly and significantly. However, negative information or eWOM makes buyers less likely to make final purchase decisions online. In this context, this study reveals several dynamic functions: (1) it emphasizes the effects on e-purchase decisions during an emergency or epidemic situation; (2) it prescribes trusted SNSs for e-purchasing; (3) it validates the technology acceptance behavior of buyers during a pandemic; and (4) it sheds light on why buyers choose a certain platform for online purchasing during an epidemic or outbreak situation. The empirical findings of this study thus inform managers and practitioners that e-purchase behavior can be significantly moderated by trust during an epidemic or pandemic crisis. Therefore, the findings suggest that marketing managers should carefully think about the marketing strategy, promotional activities, campaign-related issues, and safe delivery measures to maintain the trust factor among actual and potential online buyers. For example, Taobao, which is a popular online shopping platform in China, offered masks at lower prices to beat the “mask scammers” (Killeen, 2020). Managers and policymakers should think about the sensitive mentality of customers in relation to issues such as trust during a pandemic. Furthermore, collaboration mechanisms with such SNSs can help managers to cope with such challenging situations.

### Limitations and Future Research

The results of this research can be interpreted in light of their limitations. First, this study adopted the most commonly used theoretical model (TAM; Davis, 1989). TAM has been extensively investigated in IT acceptance. However, we utilized TAM to examine technology acceptance in a market turbulence situation. Although there are many ways to operationalize or measure a particular app’s usage behavior, we utilized the survey method, which is a classical way to conduct research. Data gathered from mobile telephone operators or from different platforms may generate a clearer picture of e-purchase behavior, although the process could be complicated. Our study focused on university students only, that is, people who are young and comfortable using technology (Hossain, Nurunnabi et al., 2020). As a result, the findings of the study are limited to a specific group of people and cannot be generalized to all of society. The respondents of the study were all students in various universities, which may also affect the generalizability of the research. Future research may use secondary data or qualitative techniques to gain deeper insights into students’ e-purchase intention during an outbreak or market turbulence situation.

Furthermore, because our conceptualization of app usage is limited to WeChat only, future research can conduct a comparative analysis among various similar apps. Finally, epidemic control situations may vary based on the fatality and infection rate, which may affect which kinds of products are most in-demand (Kampf et al., 2020). In this case, marketers may offer these necessary items as a promotional offer to retain customers. Thus, this research can be extended in the future concerning promotional measures and information-seeking. Future research may adopt a mixed-methodology approach with multi-source data, such as the dependent variable, independent variables, mediating variables, and control variables from diverse sources. Future studies may also examine the exact role of WeChat in e-purchase behavior.
